# Why eDNA fractions need consideration in biomonitoring

**DOI:** 10.1111/1755-0998.13658

**Published:** 2022-06-19

**Authors:** Magdalena Nagler, Sabine Marie Podmirseg, Judith Ascher‐Jenull, Daniela Sint, Michael Traugott

**Affiliations:** ^1^ Department of Microbiology Universität Innsbruck Innsbruck Austria; ^2^ Department of Zoology Universität Innsbruck Innsbruck Austria

**Keywords:** allochthonous DNA, ancient DNA, biodiversity assessment, environmental DNA, extracellular DNA, intracellular DNA, metabarcoding, spatial resolution, temporal resolution

## Abstract

The analysis of environmental DNA (eDNA) is revolutionizing the monitoring of biodiversity as it allows to assess organismic diversity at large scale and unprecedented taxonomic detail. However, eDNA consists of an extracellular and intracellular fraction, each characterized by particular properties that determine the retrievable information on when and where organisms live or have been living. Here, we review the fractions of eDNA, describe how to obtain them from environmental samples and present a four‐scenario concept that aims at enhancing spatial and temporal resolution of eDNA‐based monitoring. Importantly, we highlight how the appropriate choice of eDNA fractions precludes misinterpretation of eDNA‐based biodiversity data. Finally, future avenues of research towards eDNA fraction‐specific analyses are outlined to unravel the full potential of eDNA‐based studies targeting micro‐ and macro‐organisms.

## 1 eDNA AND ITS FRACTIONS

### 1.1 eDNA

**FIGURE 1:**
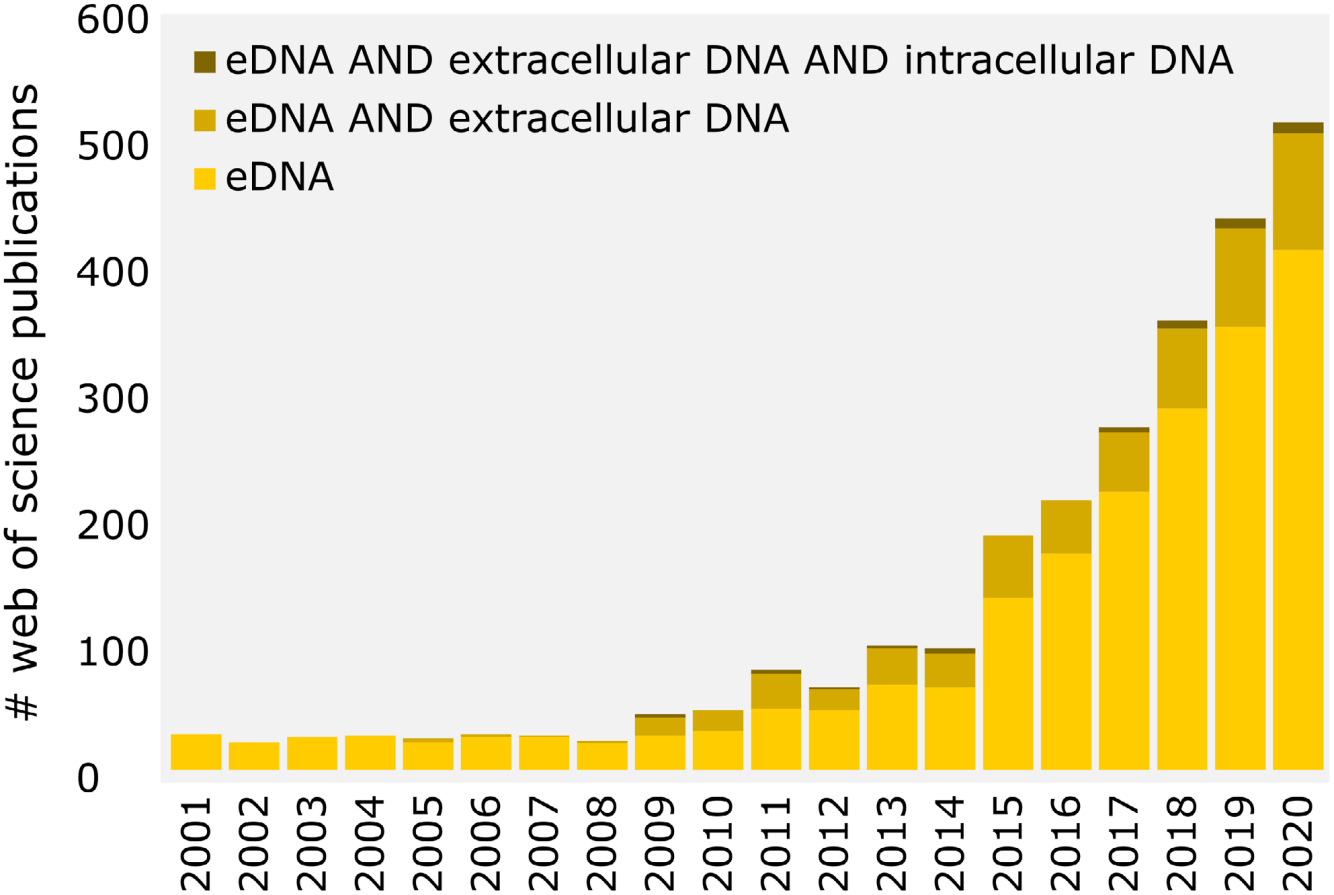
Number of articles including different search terms on webofknowledge.com between 2001 and 2020

Environmental DNA (eDNA; for expressions in bold, see Box 1) is defined as total DNA obtained from environmental samples such as water, sediment, soil or air, subsuming DNA from various sources such as unicellular or small multicellular organisms or tissue particles and gamets of multicellular organisms (Pawlowski et al., 2020). Monitoring studies based on eDNA provide comprehensive information on taxa occurring in an environment and ideally render an invasive/destructive sampling of larger organisms obsolete (e.g., diatoms, macrozoobenthos, fish, earthworms) as the target organisms do not need to be present in the sample as a whole or part. Collection of eDNA represents a versatile, easy‐to‐achieve and noninvasive approach for environmental sampling that can be standardized and effectively applied to different habitats. It enables the retrieval of information about the presence of microbial and macrobial organisms via single taxon assays or metabarcoding analyses (Cristescu & Hebert, 2018). The analysis of eDNA allows for large‐scale biodiversity assessments to identify and monitor a wide range of organisms including microbes, plants and animals with different temporal resolutions (Barnes & Turner, 2016; Deiner et al., 2017; Goldberg et al., 2016; Sepulveda et al., 2020). As such, this approach has sparked an unprecedented amount of research, as reflected by a rapidly increasing number of publications within the past two decades, but especially within the last six years (Rodríguez‐Ezpeleta et al., 2021) (Figure 1).

BOX 1

**Table:** 

**Ancient DNA (aDNA)**	DNA from ancient, extinct specimens isolated from environmental samples without defining the DNA fraction assignment
**Old DNA**	DNA from species which are not occurring in an environment during the time of sampling and which has been isolated from environmental samples without defining the DNA fraction assignment
**DNA type**	Any type of eDNA that can be further characterized by specific classification criteria. These might refer to different conformations, eDNA fractions, or DNA‐locations (plastid, mitochondrial, nuclear)
**DNA fraction**	exDNA and iDNA as fractions of the total eDNA pool, as well as f‐exDNA, wb‐exDNA and tb‐exDNA as subfractions of the exDNA but also fractions of the total eDNA pool
**dsDNA**	Double‐stranded DNA, the predominant DNA type within iDNA but less abundant within exDNA
**Environmental DNA (eDNA)**	DNA obtained from environmental samples subsuming DNA from various sources such as unicellular or small multicellular organisms or tissue particles (e.g., shed cells, faeces) and gamets of multicellular organisms, but also from different eDNA fractions, that is, exDNA and iDNA
**Extracellular DNA (exDNA)**	DNA not surrounded by intact cell wall/membrane, formed either upon release during cell lysis (after cell death) or through active extrusion by living organisms
**Free exDNA (f‐exDNA)**	Extracellular eDNA that is free in the environment, that is, not bound to any organic/mineral colloids/particles
**Low‐speed centrifugation (LSC)**	Centrifugation of ≤5000 *g*; recommended for all steps of DNA‐fractionation in order to avoid cell lysis
**Organelle DNA (oDNA)**	DNA surrounded by a double‐membrane system like in mitochondria (mtDNA of eukaryotic cells;) or plastids (ptDNA; also chloroplast DNA [cpDNAI]) found in plants, algae and some protists
**Relic DNA**	Mostly, relic DNA is used as a synonym for exDNA, assuming that it generally shows potential to persist for a long time (Carini et al., 2016; Lennon et al., 2018). We suggest, however, this term is used specifically for exDNA retrieved from samples with slow exDNA degradation rates
**Single‐stranded DNA (ssDNA)**	In soils, this type of DNA has been found to constitute the larger portion of the exDNA pool as compared to dsDNA
**Tightly bound exDNA (tb‐exDNA)**	exDNA bound to particles of the extracellular matrix or to cell membrane proteins via bivalent cations
**Weakly bound exDNA (wb‐exDNA)**	exDNA adsorbed or bound to particles of the extracellular matrix via cation bridges

**FIGURE 2:**
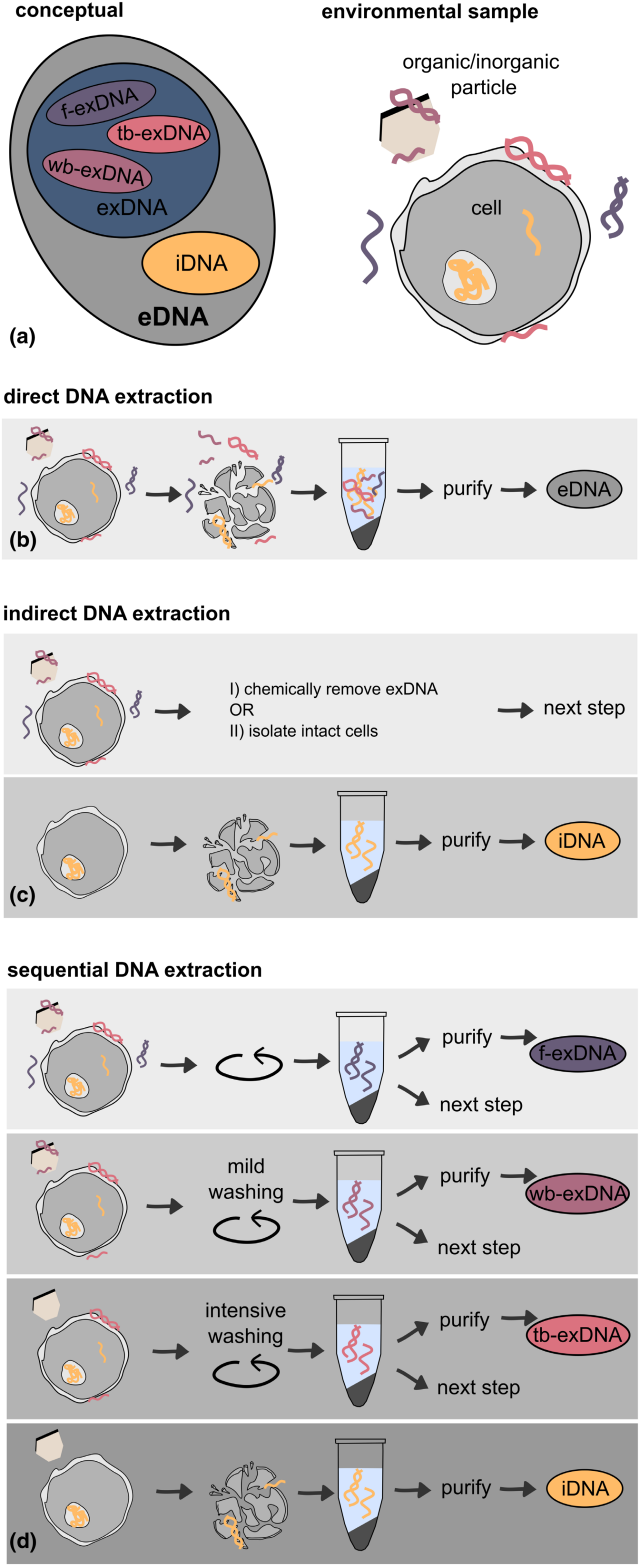
Recovery and extraction of different DNA types from environmental samples. (a) Conceptual and environmental overview how total eDNA is subdivided into its extracellular (exDNA) and intracellular (iDNA) fractions; and exDNA further into free (f‐exDNA), weakly bound (wb‐exDNA) and tightly bound exDNA (tb‐exDNA). (b), (c), and (d) conceptually depict how to obtain the different eDNA fractions (exDNA vs. iDNA) as well as differently strong bound exDNA subfractions. Depending on the type of the desired resolution level, the exDNA subfractions achieved in (d) can be pooled into one composite exDNA fraction for downstream analyses

The sum of DNA that can be extracted from an environmental sample is typically defined as eDNA and will be referred to as total eDNA in this article. Total eDNA represents a heterogeneous mixture of DNA particles that may comprise single‐stranded (ssDNA) or double‐stranded molecules (dsDNA) of genomic, mitochondrial, plastid or exosomic origin, deriving from either active and/or from inactive or lysed organisms. Moreover, and regardless of its origin or conformational character, each type of eDNA can be present inside or outside of intact cells, distinguishing two main eDNA fractions: intracellular eDNA (iDNA) and extracellular eDNA (exDNA) (Figure 2a). Here, organelle DNA (oDNA) displays a special case as it can be present outside of cells, but still be protected by an intact, double organelle membrane and thus follow similar characteristics as iDNA, or eventually after membrane‐lysis be part of the exDNA fraction. The protective nature of the mitochondrial membrane potentially slows down the decay rates of mitochondrial DNA (mtDNA) as compared to nuclear DNA (Harrison et al., 2019). Hence, the location of a DNA‐molecule within or outside of an intact cell determines its primary function, its stability and informational quality (Nagler, Insam, et al., 2018). This has led to the investigation of those fractions in different scientific fields using diverging terms (Box 2: Ambiguous definitions). However, despite the considerable interest in eDNA and, at this stage, practical implementation of the approach in biomonitoring, only about 20% of studies dealing with eDNA published from 2016 to 2020 acknowledge that eDNA consists of different fractions (Figure 1).

BOX 2 **Ambiguous definitions**

Depending on the research field, different and/or additional types of eDNA are used and rather frequently, different terms are used for the same entity. The most evident definition jumble is associated to the term eDNA: since the early 2000s, this acronym has been used to refer to extracellular DNA, particularly in biofilm‐ or microbial soil‐related studies (Steinberger & Holden, 2005) and simultaneously became a common term to refer to environmental DNA (Ficetola et al., 2008). In fact, eDNA is still frequently used to refer to both, environmental DNA or extracellular DNA (e.g., Cristescu & Hebert, 2018 vs. Pathan et al., 2020), sometimes without clearly stating whether the study refers only to the extracellular or to both fractions, leading to confusion with the risk of misinterpretation (see also the recent view by Pawlowski et al., 2020).

We propose, in accordance with the suggestion made by Cristescu and Hebert (2018), that for environmental studies “eDNA” shall be used as a standard acronym for environmental DNA only, while “exDNA” might be used to denominate extracellular DNA.

The following table gives an overview on currently used acronyms and coherent term suggestions by the authors.

**Table:** 

Suggested acronym		Alternative acronyms/identifiers
eDNA	Environmental DNA	totDNA (total DNA) (Ascher, Ceccherini, Pantani, et al., 2009; Fulgosi et al., 2012; Nagler, Podmirseg, et al., 2018; Nagler et al., 2020), tDNA (Ceccherini et al., 2009; Ramírez et al., 2018)
exDNA	Extracellular DNA	eDNA (Agnelli et al., 2007; Aldeguer‐Riquelme et al., 2021; Ascher, Ceccherini, Guerri, et al., 2009; Ascher, Ceccherini, Pantani, et al., 2009; Ceccherini et al., 2009; Gomez‐Brandon et al., 2017; Liang et al., 2021; Ramírez et al., 2018; Torti et al., 2015; Vuillemin et al., 2016; Zhang et al., 2020), extDNA (Pansu et al., 2021), relic DNA (Burkert et al., 2019;Carini et al., 2016; Lennon et al., 2018), sDNA (soluble DNA) (Lever et al., 2015), sedaDNA (sediment ancient DNA) (Haile et al., 2009)
f‐exDNA	Free extracellular DNA	fDNA (Nagler, Podmirseg, et al., 2018; Nagler et al., 2020), cfDNA (cell‐free DNA) (Gravina et al., 2016), cirDNA (Thierry et al., 2016)
wb‐exDNA	Weakly bound extracellular DNA	wbDNA (Nagler, Podmirseg, et al., 2018; Nagler et al., 2020), Wa (Pathan et al., 2020), adsDNA (adsorbed DNA) (Ceccherini et al., 2009)
tb‐exDNA	Tightly bound extracellular DNA	tbDNA (Nagler, Podmirseg, et al., 2018; Nagler et al., 2020), Ta (Pathan et al., 2020)
iDNA	intracellular DNA	nsDNA (nonsoluble DNA) (Lever et al., 2015), cellular DNA (Taberlet et al., 2012), genomic DNA (Pawlowski et al., 2020)

The aim of this opinion article is to raise awareness about the different eDNA fractions and how these fractions potentially affect eDNA‐based biomonitoring. Moreover, we provide a conceptual framework how different environmental and habitat conditions affect the presence of the different fractions (exDNA vs. iDNA) of the total eDNA pool.

### 1.2 iDNA

Environmental iDNA is present within intact cells, which may be active, dormant or dead. Being surrounded by cell walls/membranes and storing the cells' genetic information, environmental iDNA is intact and protected from enzymatic degradation, as long as the organism performs active DNA repair (Harrison et al., 2019) and/or the cell remains intact (e.g., through the formation of resting stages or within tissue particles).

### 1.3 exDNA

Environmental exDNA is formed either upon release during cell lysis (after cell death) or through active extrusion by living organisms known from bacterial, archaeal and eukaryotic organisms (Ibáñez de Aldecoa et al., 2017). Numerous functions have been attributed to exDNA, from acting as important structural component of biofilms, over mediating genetic exchange (natural transformation), to a source of nutrients, and signal of defence systems (Nagler, Insam, et al., 2018). Once released, exDNA not associated to organic or mineral particles (free exDNA, or f‐exDNA) is exposed to extracellular and cell‐associated nucleases, which are ubiquitous in soils, water and sediments (Torti et al., 2015) and are often coreleased during cell lysis. These enzymes break DNA down into smaller fragments that may bind to various particles via cation bridges (weakly bound exDNA, wb‐exDNA), or to cell membrane proteins through bivalent cations (tightly bound exDNA, tb‐exDNA) (Laktionov et al., 2004; Pathan et al., 2020). It has long been assumed that exDNA is degraded shortly after formation, being thus quantitatively irrelevant within the total eDNA pool. Growing evidence shows, however, that exDNA is abundant, quantitatively relevant, accounting for up to 60% of the total soil eDNA (Nagler, Insam, et al., 2018) and up to 90% of the total marine eDNA pool (Torti et al., 2015).

The stronger the binding of exDNA fragments to cells, organic or inorganic particles/colloids, the better its physical protection against degradation. The persistence of environmental exDNA further depends on its sequence composition, conformation (ssDNA, dsDNA) and methylation as well as on environmental conditions, where low microbial activities, low temperatures and high content of clay minerals promote a long persistence and thus detectability of exDNA (compare with temporal information) (Pietramellara et al., 2009; Zulkefli et al., 2019).

## 2 WHY DIFFERENTIATE THE FRACTIONS OF eDNA?

Directly extracting total eDNA implies that both eDNA fractions, exDNA and iDNA, are analysed simultaneously, without the possibility of further analytical discrimination. There exist, however, several methods to extract the fractions sequentially or indirectly, enabling further specific analyses, with little additional effort compared to the total eDNA‐extraction (compare with methodological approaches). If separated experimentally, these fractions potentially reveal different levels of information, while not considering them limits the output from eDNA studies and might even lead to biased or wrong conclusions in terms of species presence and abundance (compare with Box 3) (Lennon et al., 2018). Based on the scientific question, the target environment and the associated biotic and abiotic conditions, it is advisable to consider eDNA fractions separately. Thereby, exDNA and iDNA can provide specific additional information on temporal and spatial distribution (compare with temporal information, spatial information) and increase the detectability of organisms (compare with abundance and eDNA yield). This fine‐tuning DNA approach is highly cost‐efficient and robust with regard to uprising eRNA‐ and eDNA‐based methods (Cristescu, 2019; Yates et al., 2021) (compare with costs and handling benefits).

BOX 3 **Exemplary studies**

Overview on the main findings of exemplary studies comparing more than one fraction (exDNA vs. iDNA) of the total eDNA pool.

**Table:** 

Target	Study topic	Studied fractions	Main findings related to DNA fractions	Concerns	Reference
Prokaryotes (16S rRNA gene)	exDNA benthic deep‐sea ecosystems	exDNA iDNA	one third of the OTUs identified in exDNA were absent in iDNA, possibly reflecting past assemblages		Corinaldesi et al. (2018)
Bacteria Archaea (16S rRNA gene)	Potential masking effect of exDNA over iDNA in anaerobic digester	exDNA iDNA total eDNA	total eDNA renders lower species richness as iDNA; iDNA best suited for temporal community monitoring exDNA impedes detection of low abundant sequences		Nagler et al. (2021)
Bacteria Archaea (16S rRNA genes)	Sediments at different sampling core depths	exDNA iDNA	exDNA concentrations and Shannon diversities decrease with sediment sampling depth iDNA displays different trends at each site		Vuillemin et al. (2016)
Bacteria fungi	Distribution of microbiota in forest soil	exDNA total eDNA	exDNA contains information not detected in total eDNA evidences about exDNA movement throughout soil profile		Agnelli et al. (2004)
Prokaryotes fungi	Relic DNA of soil; removal via PMA	exDNA total eDNA	exDNA causes overestimation of microbial richness up to 55% when included		Carini et al. (2016)
Bacteria (16S rRNA) Eukaryotes (18S rRNA) Metazoa (COI)	Comparison of exDNA versus totDNA in aquatic sediments	exDNA total eDNA	for metazoa, observed OTU richness was higher in totDNA as compared to exDNA	exDNA protocol includes freeze–thaw step, possibly leading to iDNA → exDNA conversion	Pansu et al. (2021)
Invertebrates (18S and COI)	Soil metabarcoding for invertebrates	exDNA (phosphate buffer extraction) total eDNA	exDNA and total eDNA show major differences in eukaryotic 18S and moderate differences in COI communities >40% of species detected were unique to the two compared extraction methods (exDNA vs. total eDNA)	exDNA protocol includes freeze–thaw step, possibly leading to iDNA → exDNA conversion	Kirse et al. (2021)

### 2.1 Temporal information—The four‐scenario concept

Very few studies investigated the precise age of eDNA and its fractions, for example, via radiocarbon dating (^14^C), also because it requires large quantities of DNA and may be contaminated by “dead carbon”, as discussed by Agnelli et al. (2007). An alternative approach, utilizing eRNA to estimate the age of eDNA, was recently proposed (Marshall et al., 2021). However, in some studies, exDNA has been defined as the “old”, or relic DNA fraction (Carini et al., 2016; Fierer, 2017; Lennon et al., 2018), by assuming a longer preservation of this fraction with regard to iDNA via physical protection through binding onto organic/mineral colloids. Recent research aimed at estimating the contribution of fossil DNA to exDNA and iDNA in permafrost soils by applying a DNA repair kit to both fractions, finding that it helped to recover metagenomes from both fractions, especially in deeper layers (Liang et al., 2021). Depending on the environmental conditions, short exDNA‐fragments persisted longer in aqueous environments, enabling a better tracking of seasonal variations in community data than longer fragments (Bista et al., 2017).

Indeed, exDNA masked the information stored in iDNA if studying directly extracted total eDNA as shown for microbial sequences (Carini et al., 2016; Fierer, 2017; Nagler et al., 2021), but the finding was inconsistent as others found only minimal influence of exDNA on marine sedimentary communities (Ramírez et al., 2018). Lennon et al. (2018) modelled the fundamental processes regulating the size and composition of old exDNA pools and argued that biased estimates of biodiversity due to old exDNA are more likely if past versus recent species' abundances are distinct from one another.

In an attempt to formulate a general hypothesis for the temporal, informative character of eDNA, we propose that environments are characterized by two important constraints, namely the environmental cell lysis‐ and exDNA degradation rate. The characteristics of both rates determine how total eDNA and, more specifically, iDNA and exDNA are mirroring current local and/or past species and allochthonous eDNA input, respectively.

The specific *cell lysis rate* occurring in an environment depends on a number of factors influencing the pace at which iDNA is turned into exDNA. This can occur via apoptosis/necrosis, mechanical disruption or enzymatic degradation of cells (Harrison et al., 2019; Nielsen et al., 2007). We argue that it is probably influenced among others by osmotic pressure, pH, and microbial‐ as well as macrobial activities. However, specific relations and contributing factors require further investigation.

The *rate of exDNA degradation* is better explored: in soils, a fast decay is associated to higher temperature and moisture, high or low pH, high microbial activities and low content of clay minerals (Pietramellara et al., 2009; Sirois & Buckley, 2019). Elevated organic matter (OM) content seems to play a crucial role in trapping DNAses to soil colloids and minerals and hence, reducing the degradation speed of exDNA (Cai et al., 2006). In aquatic environments, UV‐radiation, dissolved OM‐ and salt concentrations are additional constraints potentially influencing exDNA decay (Ellegaard et al., 2020; Zhang et al., 2020). In addition, also the binding strength to particles (i.e., fDNA, wbDNA or tbDNA) determines the extent of protection, exposition and accessibility of exDNA to degradation by nucleases.

**FIGURE 3:**
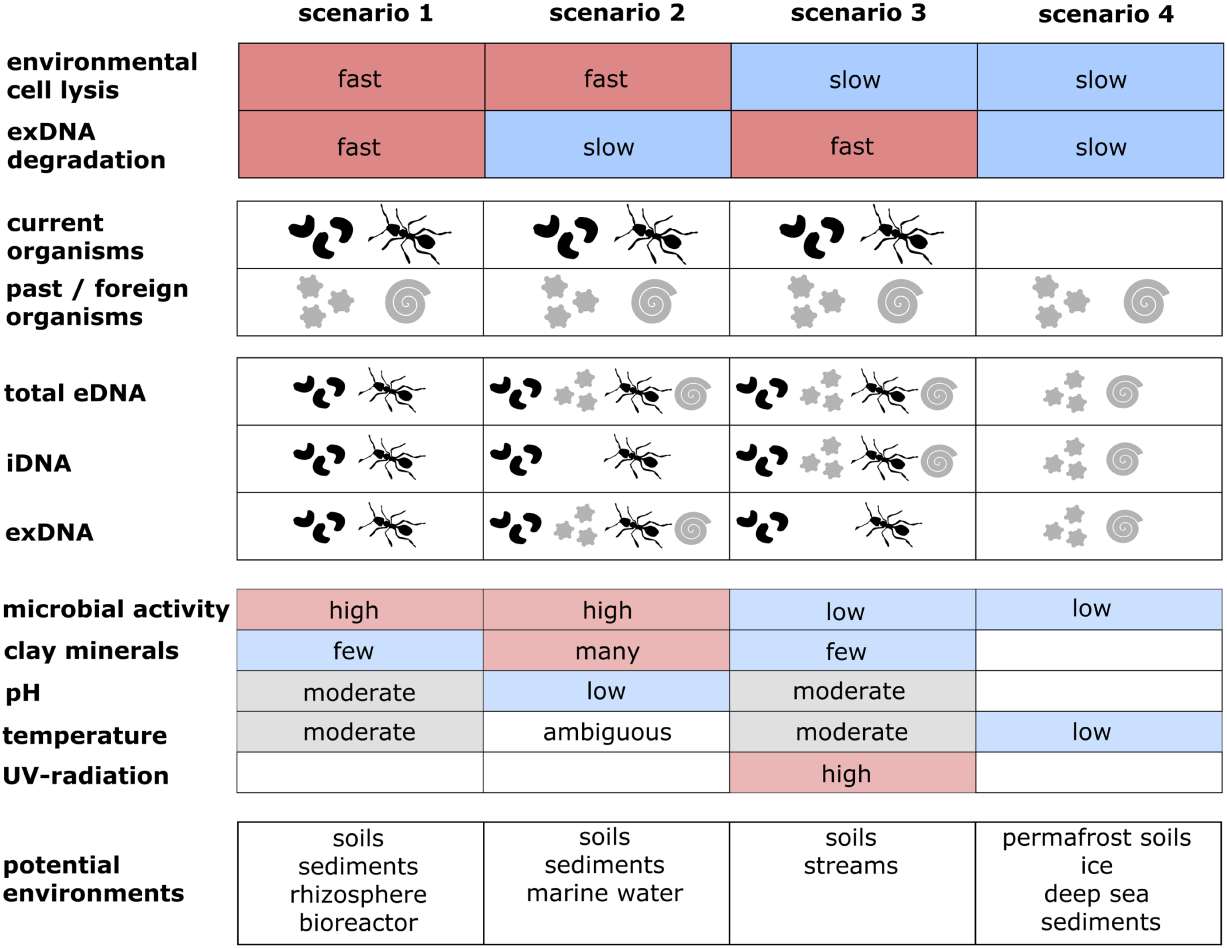
The four‐scenario concept. conceptual framework of environmental conditions governing the prevalence and persistence of eDNA fractions. Delineation of the four proposed scenarios resulting from the two environmental constraints environmental cell lysis and exDNA degradation. For each scenario, presence of organisms (black: Current vs. grey: Past/allochthonous), retrievable information on organism groups from different DNA types (total eDNA, iDNA, exDNA), potential environmental factors determining cell lysis and/or exDNA degradation rates and potential environments mirroring the properties of a scenario are given from top to bottom

Four scenarios result from the combination of the extremes of these two environmental constraints (Figure 3):

- *Scenario 1*: Fast environmental cell lysis and fast exDNA degradation. Both eDNA fractions as well as total eDNA harbour information on current organisms and are not contaminated with allochthonous/old DNA.
  Microbial hotspots such as the rhizosphere or bioreactors, but also soils and sediments with high microbial turnover and low clay mineral contents and/or other factors favouring short exDNA persistence can fulfil these criteria.
- *Scenario 2*: Fast environmental cell lysis and slow exDNA degradation. In these environments we suggest iDNA as most appropriate target to adequately study current organisms; while exDNA might contain a large amount of allochthonous or relic DNA. Studying exDNA and also total eDNA might increase the risk of false positives on currently present organisms. Under such conditions, exDNA has been defined as the old DNA fraction and was targeted to assess the distribution of past species assemblages (Corinaldesi et al., 2018; Pedersen et al., 2015).
  Scenario 2 conditions can prevail in soils or sediments with high microbial activities and thus lysis of dead cells, and at the same time a long persistence of exDNA due to for example, high content of clay minerals and/or environmental conditions that specifically preserve exDNA and protect it from degradation.
- *Scenario 3*: Slow environmental cell lysis and fast exDNA degradation. Old iDNA might accumulate within the total eDNA pool increasing the risk of false positives in iDNA, while exDNA might track current organisms and even (microbial) activities (Nagler et al., 2020; Nagler, Podmirseg, et al., 2018). exDNA from cell lysis is formed on a relatively slow rate due to the slow cell lysis rates. This is because exDNA originating from cells is rapidly degraded and the detectable exDNA is mostly deriving from cells that actively release DNA during growth. The extruded DNA is generally better protected from degradation than DNA released after cell lysis (Bylemans et al., 2018; Nagler et al., 2020; Nagler, Podmirseg, et al., 2018; Nielsen et al., 2007).
  Possible environments fulfilling these criteria are cold freshwater environments (high UV, low microbial turnover) or soils with low microbial turnover, low content of clay minerals and physicochemical conditions favouring the degradation of exDNA.
- *Scenario 4*: Slow environmental cell lysis and slow exDNA degradation. In this scenario, total eDNA as a whole decays relatively slowly. Here, eDNA is referred to as ancient DNA and can be extracted from ancient organic remains (Austin et al., 2004) such as spores, pollen and fossils (Der Sarkissian et al., 2014), making the total eDNA as well as both fractions ideal targets to study past species occurrences.
  This scenario arises under tissue‐maintaining conditions such as the deep ice or permafrost soils (Jørgensen et al., 2012).

### 2.2 Spatial information

eDNA is not stationary, but might originate from sites that are different from its sampling point. Such an allochthonous input of DNA into an environment inevitably increases the risk of false positive detections in total eDNA‐based biomonitoring studies (Ficetola et al., 2016).

Within the soil environment, exDNA can exhibit high mobility, possibly moving within the percolation water along the soil profile through leaching and force of gravity, towards the soil surface through advection by capillary force, or horizontally following the soil water flow direction (Ascher, Ceccherini, Guerri, et al., 2009). Here, ssDNA shows higher mobility than dsDNA (Pathan et al., 2020). In aquatic environments, allochthonous e(x)DNA input is a common process with implications on biomonitoring studies in running waters (Nevers et al., 2020), lakes (Vuillemin et al., 2016) and the ocean (Laroche et al., 2020).

Being surrounded by intact cells, vectors carrying iDNA are considerably larger than the naked exDNA molecule, lowering its diversion potential with regard to exDNA. However, a specific investigation on the extent of this limitation is still missing, and will certainly depend on the target organisms as well as the environment, where atmosphere and water movements might better transport microbial or single cells than shed cell aggregates or tissues. Moreover, the temporal persistence of iDNA and exDNA will influence the detectability of allochthonous DNA. Thus, we suggest that specific eDNA fractions are differently applicable to track autochthonous and/or allochthonous species under the four scenarios proposed (Figure 3).

### 2.3 Abundance and eDNA yield

Generally, abundance of harvested eDNA greatly varies with the environmental matrix, targeted species and seasonal aspects, but also with the applied DNA‐extraction approach (Stewart, 2019). As all these variables lead to incomparable results among different studies, the urgent need for method standardization is evident (Cristescu & Hebert, 2018; Pawlowski et al., 2020).

Sequential DNA extraction, that is, the discriminatory extraction of the different eDNA fractions, yields an overall higher amount of total eDNA and increased numbers of detected species than a direct extraction of the total eDNA (Ascher, Ceccherini, Pantani, et al., 2009; Nagler, Podmirseg, et al., 2018; Probst et al., 2021), reducing the risk of false negative detections. This is because consecutive sample treatment steps decrease the DNA losses that may occur during direct extraction, for example, due to a limited binding capacity of silica‐based kits. Furthermore, sequential DNA‐extraction can increase the lysis efficiency of cells in the final, iDNA yielding cell disruption/lysis step.

By investigating differences in specific eDNA fractions and determining which fraction is most representative for certain species or groups of organisms in an environment (i.e., show the strongest correlation between organismic and DNA abundance), sequential DNA extraction has the potential to minimize the error introduced by incomplete DNA‐extraction and to yield more robust estimates (Carini et al., 2016; Pansu et al., 2021; Probst et al., 2021). In addition, the organismic proxy obtainable via eDNA will benefit byreducing losses in the recovery of total eDNA through the performance of consecutive extraction steps (Ascher, Ceccherini, Pantani, et al., 2009; Nagler, Podmirseg, et al., 2018).

### 2.4 Costs and handling benefits

If exDNA is not contaminated with old or allochthonous sequences (i.e., scenarios 1 and 3; Figure 3), the sampling of exDNA represents an effective way to recover DNA from large and thus highly representative amounts of sampling material, without the need of a lysis step (e.g., soil or water) (Pansu et al., 2021; Taberlet et al., 2012). As such, this exDNA‐based assessment represents a cost‐efficient and reliable tool for large‐scale biodiversity studies over time and space (Pansu et al., 2021). However, more information on the prevalence and degradation patterns of exDNA in different environmental samples such as water, sediment and soil is needed. Likewise, also the taxon‐specific release of exDNA and iDNA into the environment is determinant for choosing the most appropriate eDNA fraction as target for specific biomonitoring purposes. This is especially true for groups of organisms (e.g., animals) which are probably secreting less exDNA into the environment compared to microbes (Dunning Hotopp, 2011).

The problem of false positives in traditional eDNA studies caused by old or allochthonous DNA is well‐known and several approaches have been proposed to meet the shortcomings (Cristescu & Hebert, 2018; Ficetola et al., 2016). A sophisticated, alternative methodology aims to overcome these issues through the use of eRNA instead of eDNA. It is supposed to be even more short‐lived than exDNA and was proposed to provide a discrete spatiotemporal signal for specific organisms (Cristescu, 2019; Yates et al., 2021). However, with regard to the more robust DNA, handling of fragile RNA is time‐ and cost‐intensive during sampling, storage and laboratory analysis and leads to a higher number of sample dropouts, ambiguous and/or less comparable results (Zaiko et al., 2018). Our proposed four‐scenario‐concept (Figure 3)—including the discriminatory analyses of exDNA and iDNA—might be capable to overcome the drawbacks of total eDNA‐based studies regarding temporal and spatial explanatory power, combining both, the accuracy of eRNA (Cristescu, 2019; Yates et al., 2021) and the cost‐efficiency and robustness of eDNA.

## 3 METHODOLOGICAL APPROACHES TO DISCERN eDNA FRACTIONS

The concept of separating the total eDNA into its extracellular and intracellular fractions (exDNA vs. iDNA) has been applied beforehand as direct versus indirect DNA‐extraction (e.g., Frostegård et al., 1999; Ogram et al., 1987). Importantly, post‐sampling shifts in the composition of the eDNA fractions should be avoided. Thus, (i) physical, chemical or enzymatic cell lysis leading to a shift from iDNA to exDNA, and (ii) the degradation of exDNA until separation of the different eDNA fractions have to be prevented. It implies avoiding sample storage in lysis‐inducing reagents, freeze–thaw cycles and high‐speed centrifugation (≥5000*g*) for fraction separation to prevent the burst of intact cells and stripping off membrane‐bound DNA particles, respectively (Aldeguer‐Riquelme et al., 2021; Peterson et al., 2012).

### 3.1 Direct eDNA‐extraction

It is the direct isolation of the total eDNA from an environmental matrix by single or combined physical, chemical or enzymatic cell lysis (Figure 2b). The resulting eDNA is a mixture of exDNA already present in the extracellular environment of the sample and iDNA, released from intact cells after cell disruption. This “total” eDNA is by far the most commonly used DNA type for eDNA‐based studies.

### 3.2 Indirect eDNA‐extraction

It is based on the recovery of intact cells from an environmental matrix through high speed centrifugation (Högfors‐Rönnholm et al., 2018) or flotation (Parachin et al., 2010), followed by cell lysis and iDNA isolation (Figure 2c). A different approach is the destructive, chemical removal of exDNA prior to cell lysis (e.g., soil [Wagner et al., 2008]; water [Hardoim et al., 2009]). Conversely, sampling methods using positively charged membrane filters (Bessey et al., 2021) or 3D‐printed hydroxyapatite samplers (Verdier et al., 2021) have been recently described as general eDNA sampling methods but might specifically catch exDNA, although further research on the recovered eDNA fraction(s) is needed.

### 3.3 Sequential eDNA‐extraction

Studies involving the explicit investigation of exDNA require a sequential eDNA‐extraction. Methods greatly vary between aqueous (Geraldi et al., 2020; Lever et al., 2015) and solid samples and include centrifugation or filter steps (Aldeguer‐Riquelme et al., 2021) (sediments [Geraldi et al., 2020; Lever et al., 2015; Ogram et al., 1987]; soil [Ascher, Ceccherini, Pantani, et al., 2009]; deadwood [Gomez‐Brandon et al., 2017]; human tissue [Laktionov et al., 2004]; anaerobic digester [Nagler, Podmirseg, et al., 2018]; water [Aldeguer‐Riquelme et al., 2021; Lever et al., 2015]). Depending on their level of resolution, sequential extractions may include one single step of exDNA‐recovery or sequential washings of the environmental sample with buffers of increasing stringency, successively yielding the different subfractions of exDNA (i.e., f‐exDNA, wb‐exDNA, tb‐exDNA)—depending on their binding strength to organic/mineral colloids or cell membranes. To collect all exDNA fractions sequentially, three steps prior to DNA purification are required (Ascher, Ceccherini, Pantani, et al., 2009; Laktionov et al., 2004; Nagler, Podmirseg, et al., 2018) (Figure 2d): free extracellular DNA (f‐exDNA) is yielded in the supernatant by washing the sample with water or alkaline sodium phosphate buffer followed by low‐speed centrifugation (LSC; i.e., ≤5000*g*) or, in case of liquid samples, by directly applying LSC. Then, wb‐exDNA is obtained by further washing the pellet with phosphate‐buffered saline containing EDTA (Nagler, Podmirseg, et al., 2018) that desorbs DNA from particles by phosphate ion competition (Torti et al., 2015) and removes ion bridges of cell‐surface‐bound DNA (Wu & Xi, 2009). LSC yields wb‐exDNA. Finally, tb‐exDNA is detached from membrane receptors via hydrolysis with trypsin and gained by LSC. The residual exDNA‐free pellet is subjected to cell disruption, and all (individual or combined) fractions are purified using commercial or in‐house purification protocols to obtain DNA compatible with downstream analyses.

## 4 CONCLUDING REMARKS

Although it is commonly accepted that total eDNA consists of different fractions (exDNA vs. iDNA), their properties and the respective consequences for the interpretation of eDNA‐based biomonitoring remain rarely acknowledged/considered, both in the analysis of eDNA samples and in the interpretation of the results. This is especially true for studies dealing with the assessment of macrobial organisms, where typically total eDNA is considered for analysis. Moreover, the currently used terminology can be misleading. Even when specific eDNA fractions are referred to as being analysed, closer inspection reveals that the findings are based on total eDNA (e.g., Bienert et al., 2012, but see Taberlet et al., 2012).

As the eDNA fractions differ in their persistence in the environment, comparative or discriminatory analyses of exDNA and iDNA might provide more detailed information about the past and present biodiversity of various habitats. The decision on which eDNA fractions shall be used for analysis should depend on (i) the type of environment sampled, in terms of environmental cell lysis rates and exDNA degradation conditions, (ii) the target organisms with their specific eDNA traits, (iii) the temporal and spatial resolution needed and, altogether, (iv) the posed research question or biomonitoring purpose. For example, exDNA has been shown to provide several advantages when monitoring microbial and macrobial species in soils and sediments (Pansu et al., 2021; Taberlet et al., 2012), whereas less is known about the pros and cons of using specific fractions of total eDNA to monitor macrobial species in aquatic environments. It is likely that for aquatic, macrobial species the assessment of which eDNA fractions are best suited for biomonitoring purposes is more complex than in sediments or soils, as the eDNA signal rapidly depicts changes in eDNA release rates, for example, during physiological and behavioural changes (Thalinger et al., 2021). Also, fragments of tissue or dead macrobial organisms might sink to the ground (dependent on water turbulences and particle size) and individual cells or exDNA be rereleased only later to the water body during decay (Curtis & Larson, 2020). Moreover, the sampling technique such as the type of filter used and the chemical properties of the sampled environment can affect the recovery success of specific eDNA fractions (Kirse et al., 2021; Liang & Keeley, 2013), and it has yet to be evaluated if filter‐based eDNA sampling methods, commonly used in aquatic systems, mainly entrap iDNA. Nevertheless, it has been shown that the pore size of filters distinctly determines the recovery rates of differently‐sized eDNA particles in fish (Jo et al., 2020), potentially driving the collection of exDNA and/or iDNA on filters. Moreover, it has been found that fish eDNA predominantly occurs as loosely aggregated tissue fragments comprising intact cells and mitochondria (Turner et al., 2014; Wilcox et al., 2015). Contrary to fish, eDNA of *Daphnia* mainly consists of subcellular eDNA of both, mitochondrial and nuclear DNA (Moushomi et al., 2019), demonstrating that the composition of eDNA fractions varies considerably between taxa. As the type of eDNA predominantly released by specific taxa drives the fate of it in different environmental settings, further research on this topic is urgently needed to optimize eDNA‐based and taxa‐focused biomonitoring (compare with Research needs).

A careful assessment of which eDNA fractions to consider will also help to minimize false positive and false negative detections. This is especially important when dealing with uncertainty in eDNA‐based decision making (Sepulveda et al., 2020). Furthermore, the choice of the specific target eDNA fraction dictates how much material from the environmental sample can be used for DNA extraction and defines the extraction method, both governing the analysis costs per sample (Taberlet et al., 2012). This in turn affects the number of samples which can be analysed within a given budget, which critically determines detection probabilities as well as spatial and temporal resolution of eDNA surveys (Pansu et al., 2021) and the overall representativeness of a survey.

Finally, in addition to the differentiation of the total eDNA pool by the type of the target environmental matrix and the taxonomic group, as suggested by Pawlowski et al. (2020), we propose a third level of eDNA description which defines the type of investigated eDNA as being either total eDNA, exDNA or iDNA. Such a differentiation is also fundamental for the ongoing creation of standards for eDNA‐based biomonitoring, a key aspect for application in routine monitoring of aquatic and terrestrial biodiversity and changes in space and time.

## 5 RESEARCH NEEDS

- Further investigate/verify where a differentiation between the eDNA fractions impacts the results for different environmental scenarios and target organisms (microbial vs. macrobial organisms); especially for macrobial organisms this information is still lacking
- Standardize sampling and DNA‐extraction: assess how physicochemical characteristics of the environmental sample (water, soil) and the storage/preservation strategy affect the extraction efficiency of the different eDNA fractions and validate/unify the sequential extraction methods for different environmental matrices
- Investigate factors influencing cell lysis rate in different environments
- Investigate how the age of total eDNA and its fractions (exDNA vs. iDNA) differ among our proposed environmental scenarios
- Assess mobility of exDNA versus iDNA in different environments

## AUTHOR CONTRIBUTIONS

Magdalena Nagler and Sabine Marie Podmirseg conceptualized the structure and content of the manuscript and wrote an initial draft. Judith Ascher‐Jenull, Daniela Sint and Michael Traugott expanded upon the ideas contained within this initial draft, and engaged in discussion and editing of the final manuscript.

## CONFLICT OF INTEREST

The authors declare no competing interests.

## Data Availability

Data availability is not applicable to this article as no new data were created or analysed in this study. Benefit sharing is not applicable to this article as no benefit was generated in this study.
